# The utilization of perfused cadaver simulation in urologic training: a pilot study

**DOI:** 10.1186/s12894-021-00895-4

**Published:** 2021-09-27

**Authors:** Daniel McClelland, Luke P. O’Connor, John Barnard, Ali Hajiran, Chad Crigger, Tyler Trump, Emma Bacharach, Amr Elbakry, Zach Werner, Chad Morley, Daniel Grabo, Adam Luchey

**Affiliations:** 1 Department of Urology, West Virginia University, 1 Medical Center Drive, PO Box 9251, Morgantown, WV 26506-9251 USA; 2 Department of Surgery, West Virginia University, Morgantown, WV USA

**Keywords:** Education, Resident, Training, Cadaver, Simulation

## Abstract

**Background:**

We sought to determine if participating in a surgical training session using perfused fresh human cadavers (PFHC) had a positive effect on urology residents’ confidence in performing open and endoscopic procedures.

**Methods:**

Urology residents at our institution participated in a surgical training session in the West Virginia University Fresh Tissue Training Program, which utilized fresh cadavers with vascular perfusion. The session consisted of performing different urologic procedures (open and endoscopic) on the perfused fresh human cadavers (PFHC). Residents were given a survey to rate their confidence in different urologic procedures before, after, and 6 months after the session. Each procedure on the survey had 3–6 questions associated with it, with scores ranging from 0 (no confidence) to 4 (great confidence). Scores for each procedure before and after the session were compared.

**Results:**

Six residents participated in the session. There was an increase in the score for every procedure performed after the session. Scores at 6 month follow up remained higher than the pre-session scores.

**Conclusion:**

PFHCs offer an excellent opportunity to teach a wide variety of urologic procedures to residents. Incorporation of PFHCs may be very useful in urologic training, and further studies on its use are warranted.

## Background

With new technologies and surgical techniques being incorporated into the practice of urology, today’s residents are tasked with learning a broader skillset than ever before. This has led to some concern that residents are not prepared for practice after completion of a 5-year surgical residency program. In fact, one study found that 23% of PGY-5 general surgery residents across 55 programs did not believe that a 5-year residency fully prepared them to practice independently [1]. In this constant-changing world of surgery, it is crucial that residency programs adapt and incorporate new methods of training to maximize residents’ readiness to practice on their own. It has been well documented that cadaver training is an effective method to enhance surgical skills by helping residents become more familiar with the anatomy and steps of surgical procedures in a low-risk setting [2–4]. In the realm of urology, studies have shown that residents believe cadaver simulation training helped them learn the steps of an operation, boosted their confidence for many procedures, and improved their surgical skills in the operating room [5]. In 2018, the West Virginia University Fresh Tissue Training Program (FTTP) was established as part of the WVU Critical Care and Trauma Institute and in partnership with the Department of Pathology, Anatomy, and Lab Medicine. The FTTP utilizes fresh, never-frozen cadavers with vascular perfusion in surgical skills training. Educational sessions take place in the FTTP lab, which includes a training room with standard operating room equipment and are moderated by surgical faculty. A case-based approach is used in surgical and procedural skills training for all levels. In this pilot study, we aim to determine if utilization of perfused fresh human cadavers (PFHC) for surgical simulation improves residents’ confidence in open/laparoscopic urologic procedures. The use of PFHCs allows residents to experience urologic procedures on full-size models where realistic adverse events, such as bleeding, can be recreated with our perfusion technology. To date, this is the first documented use of PFHCs in urology.

## Methods

### Cadaver preparation

Fresh human cadavers were obtained from the West Virginia University Human Gift Registry. All practices regarding fresh human cadaver procurement, storage, usage and removal were in accordance with the protocols and policies of the WVU Human Gift Registry and Fresh Tissue Training Program.

**Fig. 1:**
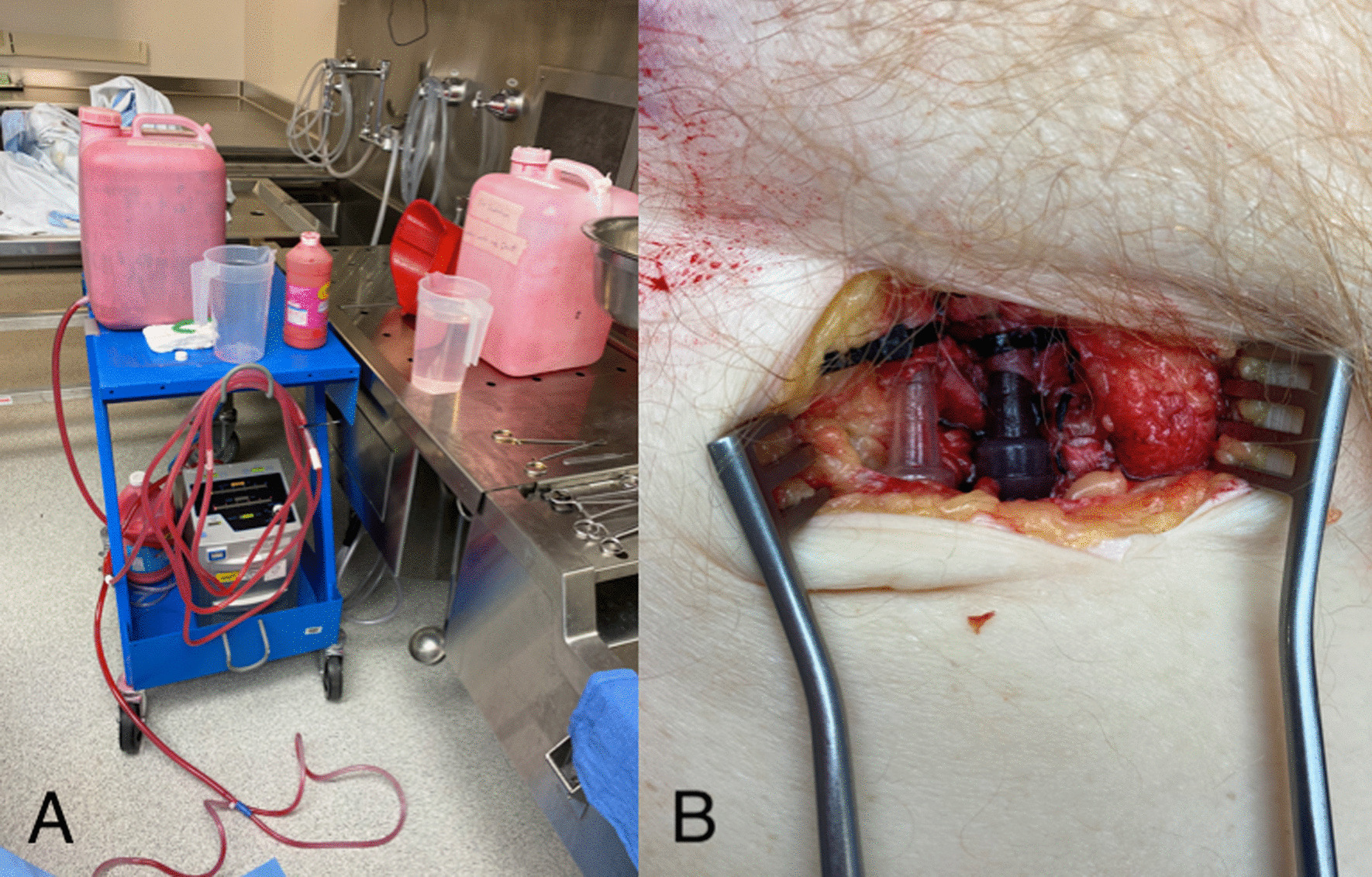
**A** Centrifugal pump set up. **B** Cannulation of femoral vessels

Fresh human cadavers used in this FTTP session were prepared in the same fashion as those previously described in the literature [2–4]. Cadavers were fresh, non-embalmed, and never frozen. They were stored in coolers maintained at 4 °C and allowed to warm at ambient temperature (20 °C) prior to scheduled training sessions. They were perfused via exposure and cannulation of the femoral vessels with Simms connectors (Fig. 1A). A centrifugal pump was used to achieve perfusate flow (Fig. 1B). Red, non-toxic paint with water was used as a blood substitute. Clamping and unclamping of the blood tubing allowed regulation of vascular perfusion [2, 3].

### Setting/participants

**Fig. 2:**
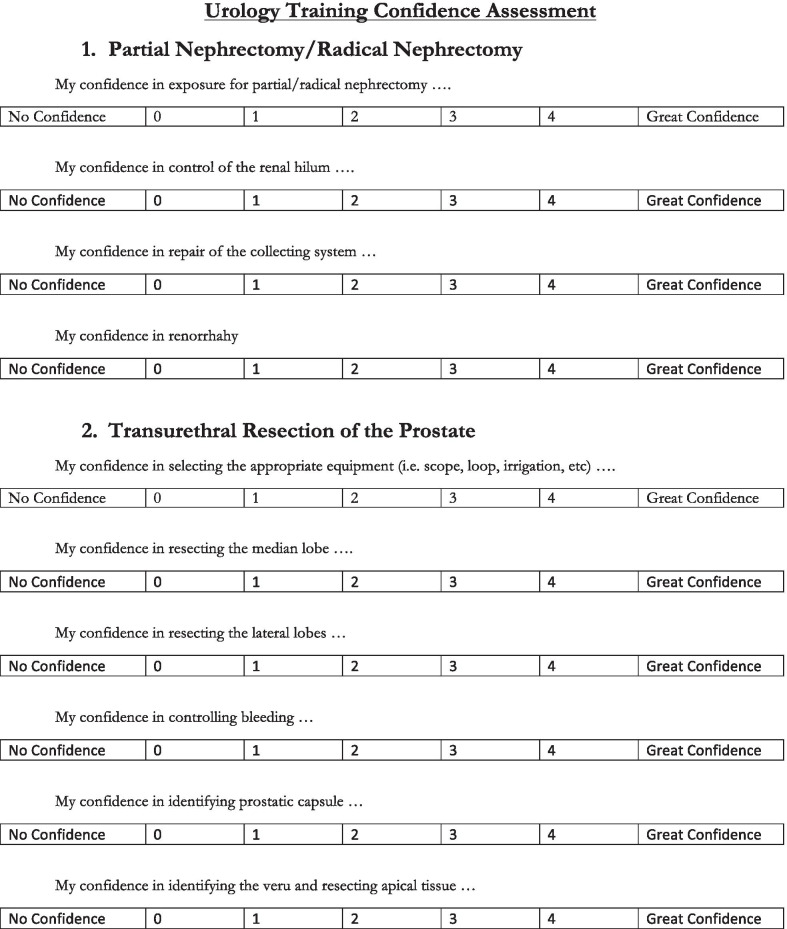
Survey questions for partial/radical nephrectomy and TURP

**Fig. 3:**
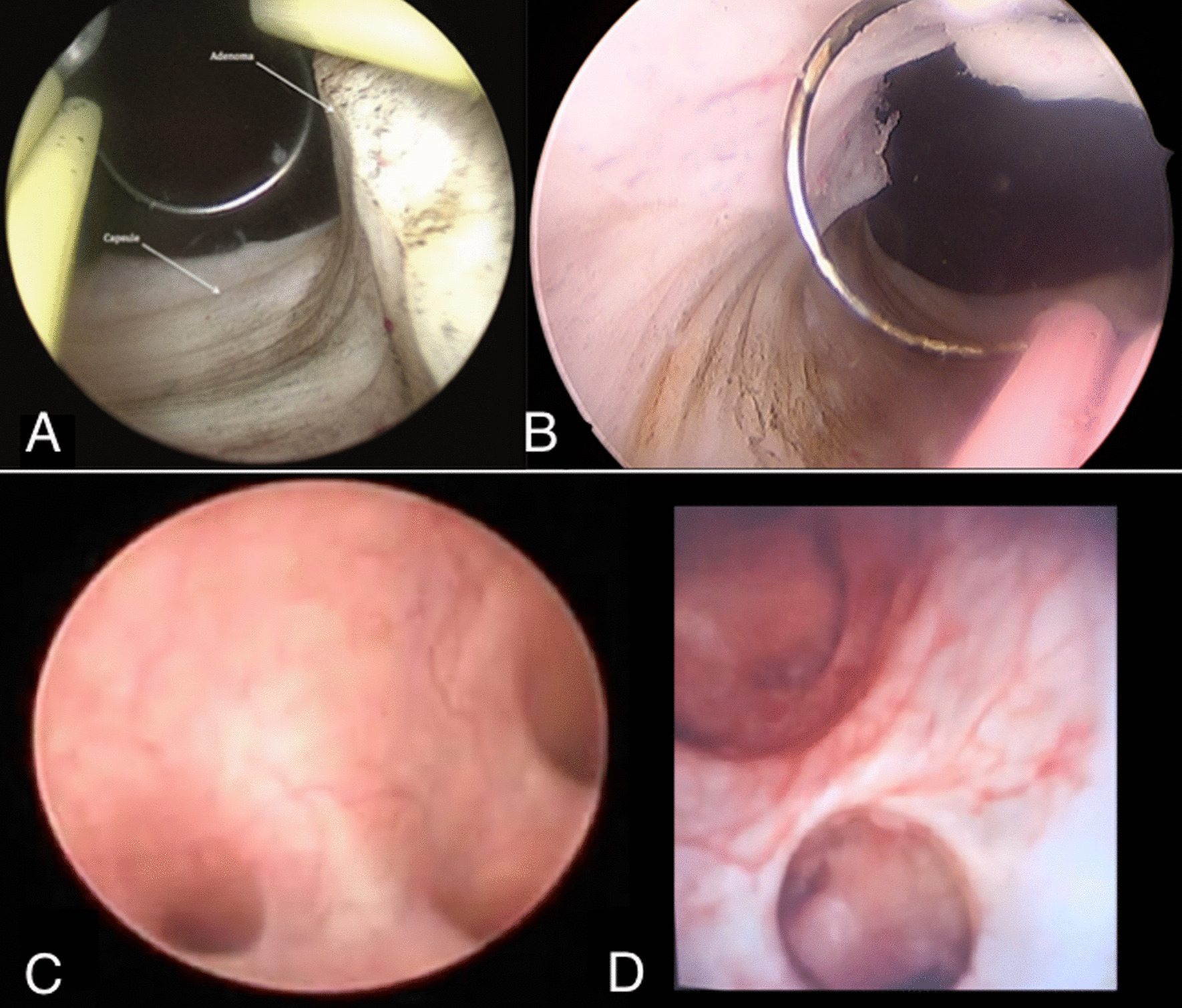
**A** TURP in a live patient. **B** TURP in a PFHC. **C** Ureteroscopy in a live patient. **D** Ureteroscopy in a PFHC

Urology residents at West Virginia University participated in a surgical skills training session in the FTTP. Residents of all levels (PGY 1 through PGY 5) participated. Prior to the start of the session, residents completed a survey that assessed confidence levels in several different urologic procedures. Each procedure on the survey had 4–6 associated questions/statements, with responses ranging from 0 (no confidence) to 4 (great confidence). For example, the section for partial nephrectomy consisted of four questions or statements (i.e. “my confidence in gaining exposure for partial nephrectomy…”), so the highest score possible for this section was 20 (as seen in Fig. 2). These procedures were then carried out in the training session under faculty supervision. Procedures performed on the PFHC model included: exploratory laparotomy, partial/radical nephrectomy, repair of ureteral/bladder injury, RPLND, groin dissection, TURBT/TURP, ureteroscopy, difficult foley catheter placement, and orchiectomy/orchiopexy. Comparisons of ureteroscopy and TURP in a live patient to a PFHC can be seen in Fig. 3. Residents then filled out the same survey immediately following the session, and again 6 months later. Residents also completed a questionnaire immediately following the session to assess their perception of the training session.

## Results

Six urology residents from our institution participated in this training session. The total scores for each procedure before the session, immediately after the session, and 6 months after the session were compared. We also evaluated the responses on the perception of the training session. When comparing scores before the session and immediately following the session, there was an increase in score for every procedure performed in all residents, with the exception of procedures in which senior residents already had a maximum score prior to the session. With outliers removed (100% confidence or no prior experience with the simulated procedure), we found that senior level residents (PGY 4 + 5) had a 9% increase in confidence scores from baseline versus 30% increase for junior level residents (PGY 1–3). For open procedures, senior level residents had a 34.1% increase in confidence level from baseline versus 37.7% for junior level residents. At six-month follow-up, scores on the survey remained higher than the pre-session scores, indicating that residents retained at least some of the knowledge and skills from the session. Scores for open and endoscopic procedures can be seen in Tables 1 and 2. Residents believed the session was a true simulation of the conditions of live surgery (100% agree or strongly agree), promoted acquisition of surgical skills (100% agree or strongly agree), and would increase their confident in handling future intra-operative consults (100% agree or strongly agree). They also believed this simulation offered benefits not available in existing training models (100% agree or strongly agree) and would recommend this training method to their colleagues (100% agree or strongly agree).

**Table 1:** Scores before, immediately following, and 6 months after session for open procedures

	Survey scores (total possible points)																	
	Exploratory laparotomy (16)			Partial/radical nephrectomy (16)			Ureteral/bladder injury repair (16)			RPLND (16)			Groin dissection (12)			Orchiectomy/orchopexy (20)		
	Before	After	6 Months after	Before	After	6 Months after	Before	After	6 Months after	Before	After	6 Months after	Before	After	6 Months after	Before	After	6 Months after
PGY-5	13	16	16	12	16	16	15	16	16	2	14	11	8	12	12	19	20	20
PGY-4	5	9	11	4	12	12	9	15	16	1	11	10	5	12	12	20	20	20
PGY-3	1	3	5	0	2	3	1	4	5	0	2	2	2	3	4	13	17	20
PGY-2a	1	3	2	0	6	4	1	6	5	1	2	2	3	6	5	10	12	18
PGY-2b	1	7	5	0	7	4	2	7	4	0	1	8	1	6	6	10	15	16
PGY-1	0	2	1	0	2	1	0	2	1	0	1	0	0	3	3	0	9	5

**Table 2:** Scores before, immediately following, and 6 months after session for endoscopic procedures

	Survey scores (total possible points)											
	Difficult foley placement (20)			TURBT (16)			TURP (24)			URS (20)		
	Before	After	6 Months after	Before	After	6 Months after	Before	After	6 Months after	Before	After	6 Months after
PGY-5	20	20	20	16	16	16	24	24	24	20	20	20
PGY-4	20	20	20	16	16	16	22	24	24	20	20	20
PGY-3	19	20	20	13	14	16	18	24	24	20	20	20
PGY-2	13	18	19	1	8	3	0	8	10	9	15	14
PGY-2	15	20	18	2	12	11	1	18	17	16	20	20
PGY-1	10	13	14	1	5	2	1	5	3	2	6	5

## Discussion

This pilot study shows that that the use of PFHCs in urologic training can be very beneficial in increasing residents’ confidence in performing a variety of urologic procedures, both open and endoscopic. Residents of all levels reported an increased score in all procedures performed. It also demonstrates that the skills and confidence gained in these sessions were at least partially retained, as scores 6 months after the session remained higher than scores prior to the session. It was noted that some scores at 6 months were higher than they were immediately following the session. This was likely due to more exposure to these procedures in the operating room within the first 6 months of completing the session. With the introduction of laparoscopy, robotics, and new minimally invasive endourologic procedures, urology residents must learn a very broad skillset during their training. Simulations such as this provide residents with extra opportunities to learn and improve their skillset in a low stress environment. Residents that participated believed the session accurately simulated the conditions of live surgery, promoted the acquisition of surgical skills, and increased their confidence of handling future intra-operative consults. Furthermore, they felt that this simulation offered benefits not available in existing training models and would recommend this training model to their colleagues.

One major advantage of PFHC over 3D printing is having the ability to operate on human tissue versus synthetic material. Many educators believe that there is truly no substitute for human tissue when learning surgical techniques [6]. 3D printing also requires highly trained computer scientists/engineers who are experts in 3D-printing. These personnel are not widely available, making it difficult for widespread implementation as there are significant barriers when starting a program as well as for maintenance of machines/software. In addition, there are significant costs to consider with 3D-printing based surgical simulation as some printers can cost > $250,000 (http://www.aniwaa.com/).

In 2012, Lewis et al. reported their experience utilizing fresh frozen cadavers to teach anatomy and operative skills to their general surgery residents [7]. Residents indicated that sessions with the cadavers were useful for learning anatomy, learning the steps of various operations, and improved their confidence in doing an operation on their own. Furthermore, the vast majority of residents expressed interest in participating in additional sessions and stated they would spend their free time in the cadaver laboratory if allowed to do so. Our study varied as we utilized fresh cadavers that never underwent a freeze/thaw cycle. In addition, junior residents were able to participate in all procedures in our simulation, even those deemed to be above their training level such as complex open procedures.

The addition of vascular perfusion further improves the validity of cadaver sessions. By using a red, non-toxic water-based paint, trainees are able to visualize bleeding in real-time during a simulated operation. Carey et al. demonstrated that mean arterial pressures of 80 mm Hg could be established, resulting in microvascular perfusion [3]. This allows for a life-like simulation for residents; bleeding will be encountered, as it would in a real surgical scenario. This type of experience is invaluable when learning how to perform minimally invasive procedures such as TURP as bleeding is one the most common complication of this procedure [8]. Encountering intraoperative bleeding on a cadaver allows residents to experience real-life complications without jeopardizing patient safety. Perfused cadavers have also been used to simulate traumatic injuries in the realm of trauma surgery. In one study, fully trained trauma surgeons participated in a session with perfused cadavers in which various injuries were simulated. Surgeons that participated reported that the perfused cadaver model allowed for an accurate simulation of the challenges faced during operative trauma and helped familiarize them with different techniques and skills [9]. While this study was primarily focused on creating a realistic training simulation, our study assessed the impact of PFHC on trainees’ confidence. While having the recreation of a realistic situation is essential, having a positive impact on trainees’ skills and confidence level is equally as important with any surgical simulation. To date, this is the first documentation of utilizing PFHCs in urologic training.

There are limitations to this study. One inherent limitation is our small sample size of 6 residents. With this small of a sample size, meaningful statistical analysis was unable to be performed. Further sessions with a larger number of urology residents need to be organized to evaluate the true impact that PFHCs may have on urologic training. Another potential limitation of this method is cost. Carey et al. reported that the average cost per PFHC was $1262.55 [3]. This may limit some training programs from using this resource depending on their funding.

The objective of this study was to introduce a new method of training to our urology residents and assess its impact on our residents’ confidence. The increase in survey scores and positive reception by our residents after just one session are very promising, and further investigation into the use of PFHCs in urologic training is warranted.

## Conclusion

This study is the first-ever documented use of perfused fresh human cadavers in urologic surgical training. This novel approach to urology training allows residents to experience the wide scope of surgical procedures and associated complications prior to real life scenarios. Further study with a larger cohort of residents is necessary to better understand the impact of perfused fresh human cadavers on urology training.

## Data Availability

The datasets used and/or analyzed during the current study are available from the corresponding author on reasonable request.
